# Colorectal cancer screening awareness among rural populations in Northern China

**DOI:** 10.1186/s12889-026-28732-z

**Published:** 2026-07-28

**Authors:** Xue Li, Feng Ji, Yang Cao, Lei Li, Liang Guan, Zheqi Zhang, Wei Cheng, Huaizhong Yang, Jian Liu, Meiji Lu, Beibei Wu, Yun Xu, Yufei Yang

**Affiliations:** 1 Department of Oncology, Xiyuan Hospital, China Academy of Chinese Medical Sciences, Beijing, China; 2 Department of Medical Biochemistry and Microbiology, Science for Life Laboratory, Biomedical Center, Uppsala University, Uppsala, Sweden; 3 Department of Oncology, The Third People Hospital of Zhengzhou, Zhengzhou, Henan China

**Keywords:** Colorectal cancer, Screening, Awareness, Risk factors, China, Rural populations

## Abstract

**Background:**

Colorectal cancer (CRC) remains one of the leading causes of cancer-related mortality worldwide, with incidence steadily increasing in China. Early detection through screening significantly reduces morbidity and mortality. However, awareness and participation remain inadequate, especially in rural populations, who account for nearly 39% of China’s total population. Understanding awareness levels and associated factors is essential for developing targeted interventions.

**Methods:**

A population-based cluster cross-sectional study was conducted between September 2022 and March 2024 in two rural villages in Beijing and Henan Province. All permanent residents were eligible for this observational assessment of CRC awareness, for this observational assessment of CRC awareness; when the number of eligible residents exceeded the required sample size, participants were randomly selected from household lists provided by local village committees to ensure proportional representation across age and sex. Data were collected using a standardized questionnaire assessing knowledge of CRC risk factors, preventive measures, early symptoms, and screening methods. Logistic regression was performed to identify factors associated with high awareness, defined as a total score ≥ 90 on the awareness scale.

**Results:**

Of the 2,091 participants, 60% demonstrated high awareness of CRC screening. Recognition of major risk factors such as advanced age (83.5%), colon polyps (88.1%), and family history (87.3%) was high, as was awareness of protective factors like dietary fiber intake/whole grains/yogurt (85.5%). Awareness of early symptoms such as blood in the stool (88.4%) and abdominal pain/incomplete evacuation (95.5%) was strong, but recognition of fatigue and anemia was lower (77.4%). Awareness of fecal occult blood testing (82.0%) and fecal genetic testing (85.1%) was slightly lower than for colonoscopy (86.7%). Logistic regression showed that age, gender, education level, alcohol consumption was significantly associated with higher awareness (*P* < 0.05).

**Conclusions:**

This study provides the first evidence of CRC awareness among rural populations in Northern China, revealing knowledge gaps in non-specific symptoms and non-invasive screening methods. Despite relatively high awareness, uptake of screening remains limited, indicating barriers such as access, cost, and cultural factors. Tailored education, accessible screening, and behavioral interventions are needed to improve prevention and early detection in these underserved communities.

**Supplementary Information:**

The online version contains supplementary material available at 10.1186/s12889-026-28732-z.

## Introduction

Colorectal cancer (CRC) is the third most commonly diagnosed cancer and the second leading cause of cancer-related mortality worldwide. In 2022, an estimated 1.93 million new cases and 930,000 deaths from CRC were reported globally, according to GLOBOCAN estimates from the International Agency for Research on Cancer (IARC) [1]. Projections indicate a substantial increase, with the global burden expected to rise by 63% to 3.2 million new cases and 73% to 1.6 million deaths by 2040. In China, CRC ranks among the top cancers, with approximately 555,000 new cases and 286,000 deaths in 2020, and projections suggesting an increase to 910,000 new cases by 2040 [2]. This underscores the urgent need for effective CRC prevention strategies and early detection efforts.

Screening for CRC plays a pivotal role in reducing both its incidence and mortality. It has been shown to lower CRC-related deaths by detecting the disease at localized stages, where five-year survival rates can exceed 90% [3]. In addition, screening allows for the removal of adenomatous polyps before they progress to malignancy, thereby effectively preventing the onset of CRC [4]. Beyond individual benefits, CRC screening holds significant public health value [5]. Widespread implementation can alleviate the economic burden associated with CRC by reducing the need for expensive late-stage treatments and enhancing population-level health outcomes [6]. Integrating CRC screening into routine healthcare services promotes health awareness, encourages preventive behaviors, and reinforces healthcare system capacity [7, 8]. Therefore, by facilitating early detection and intervention, screening programs play a critical role in reducing the global burden of CRC and advancing public health initiatives worldwide.

Although China’s economic influence has expanded, the country retains its classification as a developing nation and continues to align with the Global South in addressing shared challenges and promoting inclusive development [9, 10]. According to the National Bureau of Statistics of China, rural residents accounted for 34.9% of the total population in 2022, amounting to 491.04 million individuals [11]. This substantial proportion underscores the critical role of rural health in achieving Chinese public health objectives. Internationally, screening uptake varies widely. In high-income countries like the United States, rates among adults aged 50–75 years are around 73%, while global averages remain lower, estimated at 40–50% [12, 13]. In China, national screening rates are suboptimal, ranging from approximately 14% to 40% overall; in addition, stark disparities exist: urban areas in mega-cities like Shanghai report rates up to 40% in targeted programs, whereas rural areas lag far behind, with rates around 15–20%, reflecting gaps in infrastructure and awareness relative to international benchmarks [14, 15]. This reflects the situation in rural areas of China: despite the proven benefits of CRC screening, participation rates remain suboptimal [16]. Barriers such as limited healthcare infrastructure, financial constraints, cultural perceptions, low health literacy, and insufficient patient awareness have significantly hindered widespread screening uptake.

However, it’s worth noting that increasing public awareness of CRC screening can facilitate the use of low-cost community-level methods, such as fecal occult blood tests (FOBT) and fecal immunochemical tests (FIT), to identify high-risk populations. In addition, it can help reduce CRC risk through lifestyle modifications, including diet, alcohol consumption, and physical activity [17]. Therefore, improving the screening awareness of rural residents is essential to reduce the incidence of CRC and improve the early diagnosis rate of CRC.

Currently, in response to these challenges, China has implemented several public health initiatives, including the “Healthy China 2030” plan, which emphasizes cancer prevention and control as a national priority [18]. Despite ongoing efforts, significant disparities persist, particularly in rural areas of Northern China [19]. Understanding the gaps in awareness and knowledge regarding CRC screening in these populations is essential for designing targeted interventions that address their specific needs and barriers [20]. Therefore, this study aims to assess CRC screening awareness among rural populations in Northern China, with a focus on knowledge of risk factors, early symptoms, and available screening methods. By examining socio-demographic influences and identifying knowledge gaps, the findings of this study may help guide future public health strategies and ultimately contribute to reducing the CRC burden in rural communities.

## Methods

### Study setting and period

Beijing, the capital city of China, is one of the most populous municipalities, with a total population of approximately 21.89 million according to the Seventh National Population Census (2020) [21]. Despite its high level of urbanization, several outer districts still contain rural settlements. Taiziwu Village is a rural community located in the Miyun District of Beijing. A significant portion of Miyun District’s land consists of rural areas, and the district encompasses 2 subdistricts, 17 towns, and 1 township. As of 2022, the resident population of Taiziwu Village was approximately 3,019, based on data from Miyun District Statistical Yearbook [22].

Henan is a central province in China and the third most populous administrative region, with a total population of about 99.37 million, of whom 50.22% live in rural areas, according to the Henan Provincial Statistical Yearbook 2022 [23]. Zhaogou Village is located in Mianchi County, which falls under the jurisdiction of Sanmenxia City. Mianchi County is composed of 6 towns, 6 townships, and numerous administrative villages. As of 2022, the resident population of Zhaogou Village was approximately 1,408, according to local government records provided by the Sanmenxia City Statistical Yearbook [24].

These two sites were selected to capture regional variation: Beijing represents a highly urbanized setting with suburban-rural clusters, while Henan represents a traditional agricultural province with a high proportion of rural residents. Together, they provide insights into CRC awareness in distinct rural populations. In addition, in terms of CRC prevalence, both provinces have also reported a relatively high disease burden [25, 26].

The survey was conducted in the rural catchment areas of Taiziwu Village (Beijing) and Zhaogou Village (Henan). It was jointly organized by Xiyuan Hospital, China Academy of Chinese Medical Sciences, with support from the local village committees.

### Study design

A population-based cluster cross-sectional study was conducted among permanent residents aged 10 years and older in the two villages from September 2022 to March 2024. The two villages were treated as separate strata. Within each stratum, all eligible residents were invited to participate. When the total number of eligible residents exceeded the required sample size, participants were randomly selected from household lists provided by the local village committees to ensure proportional representation across age and sex groups.

### Study population

The study population consisted of healthy individuals of both genders residing in the selected rural areas. The inclusion of participants was primarily to obtain a comprehensive, population-representative estimate of CRC awareness across all age groups in rural communities. The study did not aim to educate minors about CRC or any adult-onset disease. The inclusion of adolescents does not imply that CRC screening awareness is expected or appropriate at this age, nor does it suggest that screening responsibility should be placed on minors. The questionnaire was presented to all participants as a general “health and lifestyle survey” without mentioning CRC or any specific cancer in the participant information sheet or assent form provided to children and adolescents. No disease-specific or potentially distressing information was disclosed to participants under 18 years of age. In addition, familial risk factors such as a family history of CRC or unhealthy lifestyle habits may already be relevant to this group, making their participation valuable [27, 28]. Besides, this study primarily selected healthy individuals and excluded participants with severe diseases, as their health perceptions and daily activities may be substantially affected by comorbidities, which could bias the assessment of awareness in the general population [29]. No disease-specific education, counseling, or screening recommendations were provided to any participants during the survey process.

### Inclusion criteria

①Individuals aged 10 years or older, regardless of gender; ②Permanent rural residents of the local area; ③Individuals with adequate communication abilities, capable of fully completing questionnaires and symptom assessment forms; ④Individuals with good compliance, willing to cooperate, and who have signed an informed consent form prior to enrollment.

### Exclusion criteria

①Diagnosed with colorectal cancer; ②Concurrent diagnosis of other malignancies or coagulation disorders; ③Presence of severe organic diseases affecting the heart, liver, kidneys, or other vital organs; ④Individuals with mental illnesses or cognitive impairments that hinder normal communication; ⑤Participants who withdraw from the study midway.

### Sampling size calculation

Sample size for this study was calculated using the following formula:

$$n=\frac{{Z}^{2}\cdot\:\mathrm{p}\cdot\:(1-\mathrm{p})\text{}}{{d}^{2}}$$

n: required sample size

Z: Z-value corresponding to the desired confidence level (for a 95% confidence level, Z = 1.96)

p: assumed proportion of the target variable (set to 0.5 for maximum variability) [30, 31]

d: margin of error (d = 0.05 for ± 5% error tolerance)

The calculation becomes:

$$\:n=\frac{{1.96}^{2}\cdot\:0.5\cdot\:(1-0.5)\text{}}{{0.05}^{2}}=384.16$$

Thus, the required sample size is approximately 385 participants. Additionally, assuming an invalid response rate of 15%, the required sample size would be 443.

### Assessment of covariates

In this study, trained researchers from Xiyuan Hospital interviewed each participant face-to-face using a standardized questionnaire developed by our research team. The following information was primarily collected. Sociodemographic variables included age, sex, marital status, and education level. Lifestyle variables included smoking consumption (yes, no) and alcohol consumption, classified as non-drinker or occasional/light drinker (participants not classified as frequent heavy drinkers) and frequent/heavy drinker (defined as drinking Chinese liquor 3 or more times per week, with at least 100 ml each time). Physical activity was categorized as regular (equivalent to brisk walking-level moderate-intensity exercise at least 3 times per week for ≥ 30 min per session, including those performing moderate or heavy manual labor) or lacking (participants not engaging in regular physical activity as defined above). Diabetes status was recorded as yes, no, or don’t know. The full English version of the questionnaire is provided in Supplementary Material 1.

### Assessment of colorectal cancer awareness

The questionnaire primarily includes the following sections: risk factors for CRC, early symptoms for CRC, screening methods for CRC, diagnostic methods for CRC, and interventions for warning symptoms. It consists of 25 questions. The questions are divided into four dimensions: Risk Factors (Questions 1–11), Protective Factors (Question 12), Early Symptoms (Questions 13–18), and Screening Methods (Questions 19–24), and Intervention Methods (Question 25).

For scoring, each correct answer to the single-choice questions earns 4 points, and question 25 is a multiple-choice question if all options are selected correctly and 2 points if fewer options are selected. The total score is 100 points, with a higher score indicating a higher level of awareness of CRC-related knowledge. In this study, a score of 90 or above is considered passing, while a score below 90 is considered failing. This threshold was determined through expert consultation and reflects a conservative approach, ensuring that only participants with near-complete knowledge across all domains were classified as having high awareness. It aligns with the principles adopted in previous questionnaire-based cancer awareness studies that favored stringent criteria.

The questionnaire is designed based on the knowledge recognized by the American Cancer Society [3]. The CRC awareness questionnaire was originally developed in Chinese by our research team and has been previously used in a study, demonstrating good reliability and validity (Cronbach’s α = 0.781, test–retest reliability = 0.845) [32]. The questionnaire was administered in its original language, as it was specifically developed for the Chinese rural population. To ensure appropriateness, a pilot test was conducted among 30 rural residents to evaluate clarity, comprehensibility, and cultural relevance, with minor modifications made accordingly. Content validity was qualitatively assessed through consultation with an expert panel, who confirmed that the questionnaire items adequately covered key aspects of CRC awareness. These steps ensured that the questionnaire was suitable for the target population. The current version was further refined for use in this study. The full English version is provided in Supplementary Material 1.

### Assessment of colorectal cancer high-risk populations

This section of the questionnaire was developed by our research team based on *the Chinese Guidelines for the Early Detection and Treatment of Colorectal Cancer (2020*,* Beijing)*, issued by the Expert Panel of the National Cancer Center [33]. The final version was established through expert consultation and consensus. The variables assessed included the following conditions: ①Family history of CRC in first-degree relatives (parents, siblings, or children); ②Personal history of colorectal polyps; ③Chronic diarrhea: defined as diarrhea lasting a cumulative total of more than 3 months over the past 2 years, with each episode lasting more than 1 week. Diarrhea is characterized by a significant increase in the frequency of bowel movements compared to usual habits (> 3 times per day), loose or watery stools with a water content > 85%, and a total daily stool weight exceeding 200 g. It is often accompanied by urgency, anal discomfort, or incontinence; ④Chronic constipation: defined as constipation lasting more than 2 months per year over the past 2 years. Constipation refers to reduced bowel movement frequency (fewer than 2–3 times per week, or only once every 2–3 days), with small, dry, and hard stools; ⑤History of chronic appendicitis or appendectomy; ⑥History of chronic biliary diseases or cholecystectomy; ⑦History of adverse life events: defined as significant life events occurring within the past 20 years that resulted in psychological trauma or distress; ⑧Positive FOBT (Fecal Occult Blood Test). The full English version of the questionnaire is provided in Supplementary Material 1.

### Statistical analysis

All statistical analyses were performed using R software (version 4.2.2). Categorical variables were summarized as frequencies and percentages, while continuous variables were presented as means with standard deviations. Differences between categorical variables were examined using the Chi-square test, and Fisher’s exact test was applied when more than 25% of cells had an expected count of less than 5.

To identify factors associated with poor CRC awareness, multivariable logistic regression was conducted with awareness level (good vs. poor) as the dependent variable. Independent variables included sociodemographic factors (age, gender, marital status, education level), lifestyle factors (smoking, alcohol consumption, physical activity), and clinical history (diabetes). All covariates were included simultaneously in the full model to adjust for potential confounders.

To ensure the validity and robustness of the logistic regression model, we assessed multicollinearity by calculating variance inflation factors (VIFs) for all factors, with all values < 10 indicating no significant collinearity. Model fit was evaluated using the Hosmer–Lemeshow goodness-of-fit test, with *P* > 0.05 indicating acceptable agreement between observed and expected outcomes. Adjusted odds ratios (ORs) with 95% confidence intervals (CIs) were reported for all factors, and a two-sided P value < 0.05 was considered statistically significant.

### Ethical approval

Ethics committee permission was obtained from the Beijing Administration of Traditional Chinese Medicine (Grant No 05-154) and Xiyuan hospital’s Ethics Review Center (Grant 2021XLA076-1). The research adhered to the ethical principles of the Declaration of Helsinki, ensuring respect for participants’ autonomy, confidentiality, and well-being. The approval explicitly encompassed the inclusion of participants aged 10–18 years. For all minors, written informed consent was obtained from their legal guardians, and assent was also obtained from the minors when appropriate.

### Ethics approval and consent to participate

Ethics committee approval was obtained from the Beijing Administration of Traditional Chinese Medicine (Grant No. 05-154) and the Ethics Review Center of Xiyuan Hospital (Grant No. 2021XLA076-1). All procedures were conducted in accordance with the ethical principles of the Declaration of Helsinki, ensuring respect for participants’ autonomy, confidentiality, and well-being.

Written informed consent to participate was obtained from all participants prior to enrollment. For participants under the age of 16, written informed consent was obtained from their parents or legal guardians. For participants aged 16–18 years, informed consent was obtained from the participants themselves, and assent was additionally obtained from minors when appropriate. The ethical approval explicitly covered the inclusion of participants aged 10–18 years.

## Results

### Characteristics of participants

The sociodemographic characteristics and specific lifestyle habits of the participants are presented in Table 1. Among the 2,091 participants who responded to the questionnaire, 51.5% were female and 48.5% were male. The majority of respondents were in the 50–60-year age group (37.4%). Regarding marital status, the majority (82.4%) were married. In terms of educational level, most participants had completed secondary school (1,324 participants, 63.3%). In addition, 1,669 participants (79.8%) were current or former smokers, whereas the majority (77.3%) did not consume alcohol. Additionally, possibly due to the fact that most participants were farmers, a large proportion (81.1%) engaged in regular physical activity, equivalent to moderate-intensity exercise such as brisk walking (≥ 3 times per week, ≥ 30 min per session), including moderate to heavy physical labor. Furthermore, most participants (82.5%) did not report having diabetes.

**Table 1:** Socio-demographic characteristics and special habits of the study participants (*N* = 2091)

Participants’ Criteria		*N* (%)
Age (years)	less than 18	45 (2.1)
	18–33	210 (10.0)
	34–49	452 (21.6)
	50–65	820 (39.2)
	66 and above	564 (27.0)
Gender	Female	1076 (51.5)
	Male	1015 (48.5)
Marital status	Single	142 (6.8)
	Married	1722 (82.4)
	Divorced	19 (0.9)
	Widow/widower	121 (5.8)
	Other	3 (0.1)
	Missing	84 (4.0)
Education level	Element school	513 (24.5)
	Secondary school	1324 (63.3)
	University	201 (9.6)
	Postgraduate studies	12 (0.6)
	Missing	41 (2.0)
Smoking consumption	Yes	1669 (79.8)
	No	413 (19.8)
	Missing	9 (0.4)
Alcohol consumption	None drinker	1616 (77.3)
	Occasionally and lightly	457 (21.9)
	Frequently and heavily	8 (0.4)
	Missing	10 (0.5)
Exercise	Often	1695 (81.1)
	Lacks	388 (18.6)
	Missing	8 (0.4)
Diabetes	Yes	151 (7.2)
	No	1724 (82.5)
	Don’t know	5 (0.1)
	Missing	211 (10.1)

For CRC-related history among the studied participants, 15 (0.7%) participants had a family history of colorectal cancer; 9 (0.4%) had a history of colorectal polyps; 40 (1.9%) had a history of chronic diarrhea; 83 (4.0%) had a history of chronic constipation; and 32 (1.5%) had a history of mucus and/or blood in their stool. Besides, 45 (2.2%) had a history of chronic appendicitis or appendectomy; 65 (3.1%) had a history of chronic cholecystitis or cholecystectomy; 68 (3.3%) had experienced any adverse life events in the past 20 years that caused emotional trauma or distress; 2 (0.1%) participants had fecal occult blood test (Supplementary Material 2, Table S1).

### Colorectal cancer screening-related awareness among study participants

**Fig. 1:**
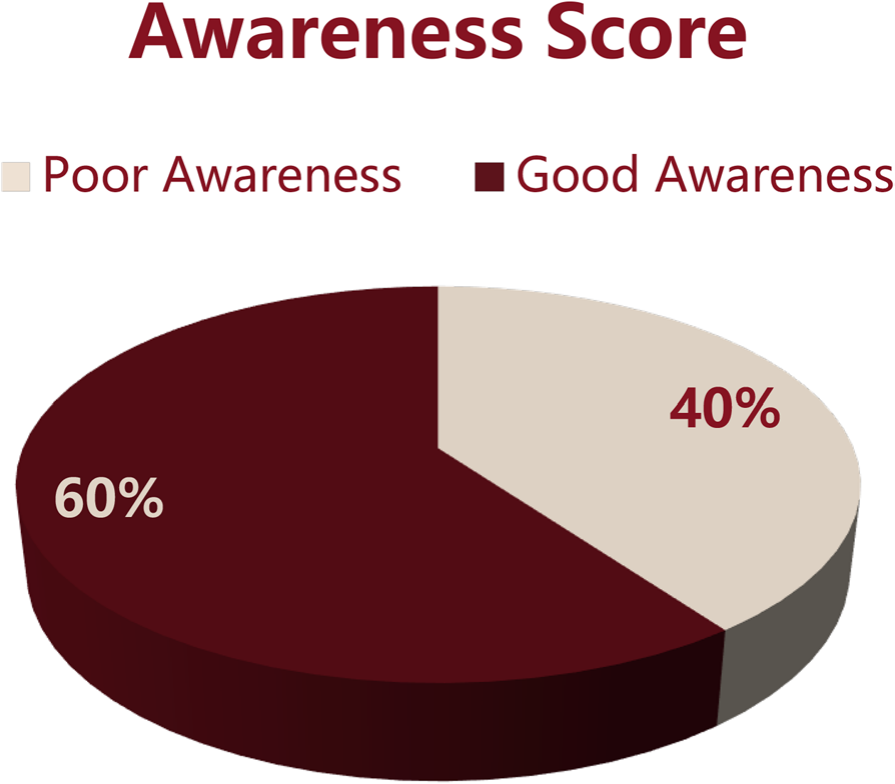
Distribution of CRC knowledge score groups among the participants (*N* = 2091)

As shown in Table 2, among the 2,091 participants, the majority recognized advanced age (83.5%), dietary risks (80.8%), smoking (81.4%), excessive alcohol consumption (83.4%), obesity (78.8%), colon polyps (88.1%), a family history of CRC (87.3%), and hereditary syndromes (88.1%) as contributing risk factors for CRC. Protective factors, such as dietary fiber intake/whole grains/yogurt, were acknowledged by 85.5% of participants. Common early symptoms, such as blood in the stool (88.4%) and abdominal pain/incomplete evacuation (95.5%), were widely recognized. However, fewer participants identified fatigue or anemia (77.4%) as early signs. Screening methods, including colonoscopy (86.7%), FOBT (82.0%), and fecal genetic testing (85.1%), were well-known. 81.4% of participants recognized colonoscopy as the gold standard. Moreover, 82.7% were aware that early-stage CRC can be asymptomatic. Furthermore, 60% of participants demonstrated a good overall awareness based on the awareness scores (Fig. 1).

**Table 2:** Colorectal cancer screening-related awareness among study participants (*N* = 2091)

Category	Variable	Question Description	Yes / Full score (%)
Risk Factors	Q1	Advanced age	83.5
	Q2	Lack of physical activity	75.7
	Q3	High consumption of meats	80.8
	Q4	Smoking	81.4
	Q5	Excessive alcohol	83.4
	Q6	Overweight/obesity	78.8
	Q7	Colon polyps	88.1
	Q8	Inflammatory bowel disease	82.5
	Q9	Family history	87.3
	Q10	Hereditary cancer syndrome	88.1
	Q11	Diabetes	76.5
Protective Factors	Q12	Dietary fiber/whole grains/yogurt	85.5
Early Symptoms	Q13	Rectal bleeding	86.1
	Q14	Blood in the stool	88.4
	Q15	Chronic diarrhea	86.2
	Q16	Fatigue/anemia	77.4
	Q17	Abdominal pain/incomplete evacuation	95.5
	Q18	Asymptomatic early-stage	82.7
Screening Methods	Q19	Colonoscopy	86.7
	Q20	Fecal occult blood test (FOBT)	82.0
	Q21	High-risk questionnaire	79.3
	Q22	Fecal genetic testing	85.1
	Q23	Digital rectal examination	79.2
	Q24	Colonoscopy as gold standard	81.4
Intervention Methods	Q25	Prevention measures	22.8

Note: Missing responses were < 1% for most items

Table 3 summarizes the distribution of awareness levels by sociodemographic characteristics, lifestyle factors, and CRC-related risk factors. Significant associations were observed between CRC-screening awareness and participants’ age (*P* < 0.0001), marital status (*P* = 0.0004), smoking (*P* < 0.0001), alcohol consumption (*P* < 0.0001), history of chronic diarrhea (*P* = 0.005), chronic constipation (*P* = 0.0002), mucus and/or blood in stool (*P* = 0.008), chronic appendicitis or appendectomy (*P* = 0.044), chronic cholecystitis or cholecystectomy (*P* = 0.0005), and fecal occult blood test results (*P* = 0.0009). However, a family history of CRC and experiences of major emotional trauma were not significantly associated (*P* > 0.05). The full table is shown in Supplement Material 2, S2.

**Table 3:** Comparison of study participants’ awareness score groups regarding their sociodemographic characteristics, special habits, and CRC-related risk factors

		Awareness		*p*-value
		Poor (*N* = 838)	Good (*N* = 1253)	
Age (years)	less than 18	9 (1.0)	27 (2.1)	< 0.0001**
	18–33	63 (7.5)	147 (11.7)	
	34–49	162 (19.3)	290 (23.1)	
	50–65	358 (42.7)	462 (36.9)	
	66 and above	246 (29.4)	318 (25.4)	
Marital status	Single	42 (5.0)	100 (8.0)	0.0004**
	Married	723 (86.3)	1083 (86.4)	
	Divorced	13 (1.6)	6 (0.5)	
	Widow/widower	57 (6.8)	64 (5.1)	
	Others	3 (0.4)	0 (0.0)	
Smoking consumption	Yes	211 (25.2)	202 (16.1)	< 0.0001*
	No	627 (74.8)	1051 (83.9)	
Alcohol consumption	None drinker	571 (68.1)	1055 (84.2)	< 0.0001**
	Occasionally and lightly	265 (31.6)	192 (15.3)	
	Frequently and heavily	2 (0.2)	6 (0.5)	
Do you have a history of chronic diarrhea?	Yes	25 (3.0)	15 (1.2)	0.005*
	No	813(97.0)	1238(98.8)	
Do you have a history of chronic constipation?	Yes	50(6.0)	33(2.6)	0.0002*
	No	788(94.0)	1220(97.4)	
Do you have a history of mucus and/or blood in your stool?	Yes	5(0.6)	27(2.2)	0.008*
	No	833(99.4)	1226(97.8)	
Do you have a history of chronic appendicitis or appendectomy?	Yes	11(1.3)	34(2.7)	0.044*
	No	827(98.7)	1219(97.3)	
Do you have a history of chronic cholecystitis or cholecystectomy?	Yes	12(1.4)	53(4.2)	0.0005*
	No	826(98.6)	1200(95.8)	
What were the results of your fecal occult blood test (immunological method)?	Yes	0(0.0)	2(0.2)	0.0009**
	No	20(2.4)	66(5.3)	
	Others	818(97.6)	1185(94.6)	

*Chi-squared test, Bold *p*-values indicating significance

**Fisher Exact test, Bold *p*-values indicating significance

Table 4 presents the results of the multivariable logistic regression analysis examining factors associated with poor awareness regarding CRC screening among study participants. After adjustment for potential confounders, age, gender, education level, and alcohol consumption habits were significantly associated with poor awareness. Older age groups were associated with poor awareness, with ORs of 0.448 (34–49 years), 0.323 (50–65 years), and 0.323 (≥ 66 years), while the 18–33 age group was not significantly different (OR: 0.583). Being male was significantly associated with poor awareness compared to females (OR: 1.455, 95%CI: 1.165–1.821, *P-value* = 0.001). Higher education levels, particularly university or tertiary education, were strongly associated with reduced odds of poor awareness (OR: 0.210, 95% CI: 0.058–0.705, *P* = 0.012). In addition, occasional alcohol consumption was associated with lower odds of poor awareness compared to abstinence (OR: 0.380, 95%CI: 0.286–0.502, *P* < 0.001). Model diagnostics indicated acceptable fit (Hosmer–Lemeshow test, *P* = 0.421) and no evidence of multicollinearity (all VIF < 2), suggesting robustness of the results. Sensitivity analysis excluding participants younger than 18 years yielded results largely consistent with the primary analysis (Supplementary Material 2, Table S4). A minor change was observed for the primary education level, where the P value shifted from borderline non-significance to statistical significance. This change is likely attributable to alterations in sample composition and the small number of participants in this subgroup, rather than a substantive change in the underlying association.

**Table 4:** Factors associated with CRC-screening poor awareness among study participants (*N* = 2091)

		B	Exp(B)	95% C.I for EXP(B)		*p*-value
				Lower	Upper	
Age (years)	less than 18					
	18–33	-0.539	0.583	0.265	1.283	0.180
	34–49	-0.804	0.448	0.210	0.952	**0.037**
	50–65	-1.131	0.323	0.153	0.678	**0.003**
	66 and above	-1.130	0.323	0.153	0.684	**0.003**
Gender	Female					
	Male	0.375	1.455	1.165	1.821	**0.001**
Marital status	Single					
	Married	0.058	1.060	0.614	1.807	0.833
	Divorced	-0.965	0.381	0.115	1.164	0.099
	Widow/widower	-0.262	0.770	0.395	1.488	0.438
	Others	-13.749	0.000	--	--	0.959
Education level	No formal education					
	Primary level	-0.217	0.805	0.635	1.018	0.071
	Secondary level	-0.346	0.708	0.527	0.950	**0.022**
	College level	-0.903	0.405	0.269	0.609	**0.000**
	University/Tertiary level	-1.560	0.210	0.058	0.705	**0.012**
Smoking consumption	Yes	-0.263	0.769	0.575	1.031	0.078
	No					
Alcohol consumption	None drinker					
	Occasionally and lightly	-0.969	0.380	0.286	0.502	**0.000**
	Frequently and heavily	0.405	1.500	0.332	10.511	0.627
Exercise	Often	-0.164	0.849	0.668	1.075	0.176
	Lacks					
Diabetes	Yes	0.060	1.062	0.749	1.515	0.736
	No					

B the unstandardized regression coefficient; Exp(B) the exponentiated coefficient, indicating the adjusted odds ratio; 95% C.I. the 95% confidence interval

## Discussion

The global burden of CRC continues to rise, especially in low- and middle-income countries, where access to medical services is limited, leading to delayed diagnosis and poor clinical outcomes [34]. This underscores the urgent need for effective CRC prevention strategies and early detection efforts. Our study underscores the urgent need for effective CRC prevention strategies and early detection efforts, which provides novel insights into CRC awareness among rural populations in Northern China, marking the first investigation of its kind. CRC is predominantly a disease of later adulthood and that organized screening initiatives should primarily target age-appropriate populations. This study recruited younger participants to provide a comprehensive community-level overview of rural households’ fundamental health knowledge, rather than to suggest individual-level accountability or expected knowledge standards. The inclusion of younger participants in cancer awareness surveys has also been reported in previous international studies assessing baseline cancer knowledge at the population level, particularly when the objective was descriptive rather than interventional [35, 36]. Consistent with this rationale, sensitivity analyses excluding participants under 18 years confirmed that the main findings were robust, with only minor variations observed in low-frequency subgroups. Conducted with 2,091 participants aged 10–96 years, with diverse educational backgrounds and a near-equal gender distribution, the study found that 60% of participants demonstrated good CRC awareness. This level is relatively higher than in comparable studies, such as a nationwide survey in Egypt (one-third awareness) and a Lebanese study reporting low awareness and screening adherence [37, 38].

It is important to note that CRC incidence differs across these regions. In China, the age-standardized incidence rate of CRC is approximately 23.9 per 100,000 persons, reflecting a rising burden in urban and rural areas [39]. In Egypt, the incidence is lower, estimated at 12.1 per 100,000, while Lebanon reports a rate of 16.3 per 100,000 [40, 41]. These differences may partly account for variations in awareness. The higher disease burden in China, combined with public health initiatives such as Healthy China 2030, is likely to foster greater emphasis on CRC education. By contrast, in Egypt and Lebanon, the lower prevalence may diminish the perceived urgency. The relatively high awareness observed in our study, conducted among 2,091 participants in rural Northern China, may reflect the local impact of public health initiatives like Healthy China 2030 and the role of rural healthcare networks and digital platforms in disseminating health information. However, given the study’s limited sample size relative to China’s large population, these findings may not be fully representative of all rural regions in Northern China.

Although awareness levels were relatively high, actual participation in CRC screening was extremely low in our study, with only 0.1% reporting FOBT. However, according to data from the U.S. NHIS, approximately 9.3% of U.S. residents underwent FOBT screening in 2023. Such data is not satisfactory [42]. This gap underscores that awareness alone does not translate into screening behavior. Despite relatively high CRC awareness, systemic barriers such as limited healthcare access, financial constraints, and cultural stigmas continue to impede screening participation in rural China [43–45]. These findings suggest that awareness alone is insufficient to drive screening uptake. Drawing from international best practices, integrating non-invasive screening methods and community outreach programs could address these barriers.

### Colorectal cancer risk factors

Participants demonstrated strong awareness of major CRC risk factors, such as advanced age, colon polyps, and family history, as well as protective factors like dietary fiber intake. This aligns with findings from other studies and suggests that health education initiatives have effectively communicated critical aspects of CRC to rural populations [46, 47]. Similarly, high recognition of early symptoms, particularly blood in the stool and abdominal pain/incomplete evacuation, underscores the potential for targeted public health campaigns to enhance early detection. While abdominal pain is generally considered a nonspecific manifestation, some studies have reported it as an early presentation of CRC, especially in right-sided tumors [3, 48]. Future research should distinguish between them to more accurately capture public awareness.

However, gaps in awareness persist. Participants remained unable to identify some non-specific symptoms like fatigue or anemia, which may delay diagnosis. Additionally, lower recognition of lifestyle-related risk factors, such as lack of physical activity and diabetes, indicates an incomplete understanding of CRC’s multifactorial etiology. This knowledge gap could hinder the adoption of comprehensive preventive strategies, especially in rural areas where access to healthcare is limited.

Awareness of less invasive screening modalities, such as high-risk questionnaires and digital rectal examinations, lags behind that of colonoscopy. This disparity may discourage screening participation in rural settings with limited access to advanced medical facilities, exacerbating health inequities [49]. While fecal occult blood tests and fecal genetic testing are relatively well-known, their limited availability in rural areas underscores the need for broader access to cost-effective screening options [50]. Public health strategies should prioritize promoting these alternatives and addressing knowledge gaps through targeted education to enhance CRC prevention and early detection in underserved populations.

### Factors influencing awareness of colorectal cancer

Several sociodemographic characteristics, lifestyle factors, and CRC-related risk factors differed across awareness groups. CRC-screening awareness varied by sociodemographic characteristics, lifestyle habits, and gastrointestinal disease histories. These findings suggest that personal characteristics and health experiences may influence awareness, consistent with previous research. After adjusting for potential confounders in logistic regression, older age, female, university or tertiary education, and occasional alcohol consumption were significant independent factors associated with good awareness.

These findings are consistent with previous studies indicating that men generally exhibit lower awareness of CRC and other cancer screenings compared with women, likely reflecting differences in health-seeking behaviors and preventive care engagement [51]. The strong protective effect of higher education aligns with evidence that educational attainment is a key determinant of health literacy and preventive health knowledge [6, 52–54]. The observed association between occasional alcohol consumption and higher awareness may reflect greater social engagement and exposure to health information, a pattern noted in some population-based surveys [55]. Older age was associated with better awareness of CRC screening, likely reflecting greater health-seeking behavior and exposure to health education over the lifespan [37–57]. Overall, our results underscore the need for targeted educational strategies focusing on men and individuals with lower education, as well as leveraging routine healthcare encounters to improve CRC screening awareness and, ultimately, participation.

### Future research priorities

#### Behavioral insights into screening participation

Despite 60% of participants demonstrating high awareness of CRC risk factors, protective factors, and early symptoms, translating this knowledge into screening participation remains challenging. Future research should investigate psychological, cultural, and systemic barriers to screening uptake using behavioral economics frameworks or qualitative methods to uncover decision-making processes [58]. This will help design interventions that effectively convert awareness into action.

#### Cost-effective screening strategies

Although participants have a relatively high awareness of CRC screening, lower adoption of non-invasive, cost-effective screening methods like fecal occult blood tests and fecal genetic testing, which are viable alternatives to colonoscopy [59]. These methods are more affordable and acceptable to individuals hesitant about invasive procedures. This phenomenon also indicates to a certain extent that cognition does not necessarily drive action. Given the findings of this study, future research should replicate these investigations in larger, more diverse rural populations across Northern China to confirm the generalizability of our results. Studies should focus on addressing systemic barriers (e.g., cost, accessibility, and cultural stigmas) and evaluating scalable, cost-effective screening programs tailored to rural settings to enhance CRC screening uptake.

#### Tailored interventions for specific demographics

Awareness gaps persist among male and those with lower education levels. Leveraging digital platforms, such as mobile health (mHealth) interventions, social media, or telemedicine, could enhance CRC screening uptake in rural areas with widespread smartphone use. Research should assess the effectiveness of these platforms and community-led initiatives, including empowering women as health influencers to amplify awareness campaigns. Additionally, the lack of correlation between awareness and family history of CRC suggests a need to explore misconceptions about hereditary risks through targeted education and genetic counseling programs.

In summary, the study underscores the role of socioeconomic status and education in influencing CRC awareness. Further investigations should focus on structural factors contributing to these disparities and evaluate the impact of policy interventions, such as subsidized screenings or improved rural healthcare infrastructure, in mitigating these inequities. In addition, although this study provides only a snapshot of CRC awareness, longitudinal research is needed to assess changes in awareness and the impact of awareness on screening behaviors and health outcomes This will help assess the long-term effectiveness of public health campaigns like Healthy China 2030.

### Limitations

The cross-sectional nature of the study limits its ability to establish causal relationships between awareness levels and actual screening behaviors. Longitudinal studies are needed to assess the long-term impact of awareness on CRC prevention outcomes. Second, participant recruitment was facilitated through local village committees, which may have led to an over-representation of health-conscious individuals. Consequently, the overall awareness level observed in this study may be slightly overestimated compared with the general rural population. Third, in our questionnaire, some symptoms were combined into a single item. Combining these symptoms may reduce accuracy, and future surveys should separate them. While the study focuses on rural populations in Northern China, the findings may not be generalizable to other regions of China, especially areas with different socioeconomic, cultural, or healthcare contexts. Additionally, the study’s sample size of 2,091 participants, while diverse in age and educational background, is relatively small compared to China’s population of over 1.5 billion. This limits the generalizability of our findings to other rural regions in Northern China or beyond, necessitating larger-scale studies to validate and expand upon these results. Finally, although adolescents constituted a very small proportion of the sample (2.1%), future studies may focus exclusively on age-eligible screening populations to further align with screening policy frameworks.

## Conclusion

This study provides the first evidence of CRC awareness among rural populations in Northern China, showing that while overall awareness is relatively high, significant gaps persist in knowledge of non-specific symptoms and non-invasive screening methods. Awareness alone does not translate into screening participation, highlighting systemic barriers such as limited healthcare access, cost, and cultural factors. Future efforts should focus on tailored educational interventions, cost-effective and accessible screening strategies, and behavioral approaches to convert awareness into action. Longitudinal studies are needed to evaluate the sustained impact of public health initiatives and inform strategies for improving CRC prevention and early detection in underserved rural populations.

**Competing interests**

The authors declare no competing interests.

## Supplementary Information

Supplementary Material 1.

Supplementary Material 2.

## Data Availability

The datasets used and/or analyzed during the current study are available from the corresponding author on reasonable request.

## Abbreviation

CRC: Colorectal cancer
