# Supplementary material for: Colorectal cancer screening awareness among rural populations in Northern China
Source: BMC Public Health. 2026 Jul 28;26:2234. doi: 10.1186/s12889-026-28732-z (PMC13418789; doi:10.1186/s12889-026-28732-z)
Supplement: Supplementary file 1 — Supplementary Material 1. [file 12889_2026_28732_MOESM1_ESM.docx]

**Colorectal cancer screening awareness among rural populations in Northern China**

**Demographic Questions**

**1.** **What is your age (years)? □□**

**2. What is your gender?**

**□** Female

**□** Male

**3. What is your marital status?**

**□** Single

**□** Married

**□** Discovered

**□** Widow/widower

**□** Other

**4. What is the highest level of education qualification you have obtained?**

**□** Element school

**□** Secondary school

**□** University

**□** Postgraduate studies

**5. Do you currently smoke or have you smoked in the past?**

**□** Yes

**□** No

**6. Do you currently drink alcohol or have you in the past?**

**□** None drinker

**□** Occasionally and lightly (Participants not classified as frequent heavy drinkers)

**□** Frequently and heavily (Drinking Chinese liquor 3 or more times per week, with at least 100 ml each time)

**7. Do you engage in** **physical activity in your daily life?**

**□** Often (Equivalent to brisk walking-level moderate-intensity exercise, at least 3 times per week for ≥30 minutes per session; includes those performing moderate or heavy manual labor)

**□** Lacks (Participants classified as not engaging in regular physical activity as defined above)

**8. Have you ever been diagnosed with diabetes?**

**□** Yes

**□** No

**Questions on High-Risk Factors for Colorectal Cancer**

**1. Have any of your first-degree relatives (including parents, siblings, or children) been diagnosed with colorectal cancer?**

□ Yes

□ No

**2. Have you ever had or currently have colorectal polyps?**

□ Yes

□ No

**3. Have you ever had or currently have chronic diarrhea?** (Defined as diarrhea lasting a cumulative total of more than 3 months over the past 2 years, with each episode lasting more than 1 week. Diarrhea is a common symptom characterized by a significant increase in the frequency of bowel movements compared to usual habits (>3 times per day), loose or watery stools with a water content >85%, and a total daily stool weight exceeding 200 grams. It is often accompanied by a sense of urgency, anal discomfort, or incontinence.)

□ Yes

**□** No

**4. Have you ever had or currently have chronic constipation?** (Defined as constipation lasting more than 2 months per year over the past 2 years. Constipation refers to reduced bowel movement frequency—fewer than 2–3 times per week, or only once every 2–3 days—with small, dry, and hard stools.)

**□** Yes

**□** No

**5.** **Have you ever had or currently have stools containing mucus and/or blood?**

**□** Yes

**□** No

**6. Have you ever experienced chronic appendicitis or undergone an appendectomy?**

**□** Yes

**□** No

**7. Have you ever had or currently have chronic cholecystitis or cholecystectomy?**

**□** Yes

**□** No

**8. Over the past two decades, have you experienced adverse life events leading to psychological trauma or suffering (e.g., divorce, death of spouse or close family member, job loss)?**

**□** Yes

**□** No

**9. Was your FOBT (Fecal Occult Blood Test) positive?**

**□** Yes

**□** No

**□** Undetected

**Questions on Colorectal Cancer Awareness**

**1. Is advanced age a risk factor for colorectal cancer?**

□ Yes

**□** No

**2. Is lack of physical activity a risk factor for colorectal cancer?**

**□** Yes

**□** No

**3. Is high consumption of beef, lamb, and processed meats a risk factor for colorectal cancer?**

**□** Yes

**□** No

**4. Is smoking a risk factor for colorectal cancer?**

**□** Yes

**□** No

**5. Is excessive alcohol consumption a risk factor for colorectal cancer?**

**□** Yes

**□** No

**6. Is overweight or obesity a risk factor for colorectal cancer?**

**□** Yes

**□** No

**7. Are colon polyps a risk factor for colorectal cancer?**

**□** Yes

**□** No

**8. Is inflammatory bowel disease a risk factor for colorectal cancer?**

**□** Yes

**□** No

**9. Is a family history of colorectal cancer a risk factor for colorectal cancer?**

**□** Yes

**□** No

**10. Is hereditary cancer syndrome a risk factor for colorectal cancer?**

**□** Yes

**□** No

**11. Is diabetes a risk factor for colorectal cancer?**

**□** Yes

**□** No

**12. Is the intake of dietary fiber, whole grains, and fermented dairy products (such as yogurt) a protective factor against colorectal cancer?**

**□** Yes

**□** No

1. **Is rectal bleeding with bright red blood an early symptom of colorectal cancer?**

**□** Yes

**□** No

**14. Is blood in the stool, which might make the stool look dark brown or black, an early symptom of colorectal cancer?**

**□** Yes

**□** No

**15. Is chronic diarrhea an early symptom of colorectal cancer?**

**□** Yes

**□** No

**16. Is fatigue or anemia an early symptom of colorectal cancer?**

**□** Yes

**□** No

**17. Is abdominal pain or a feeling of incomplete bowel evacuation an early symptom of colorectal cancer?**

**□** Yes

**□** No

**18. Can early-stage colorectal cancer be asymptomatic?**

**□** Yes

**□** No

**19. Is colonoscopy a screening method for colorectal cancer?**

**□** Yes

**□** No

**20. Is the fecal occult blood test a screening method for colorectal cancer?**

**□** Yes

**□** No

**21. Is a high-risk questionnaire a screening method for colorectal cancer?**

**□** Yes

**□** No

**22. Is fecal genetic testing a screening method for colorectal cancer?**

**□** Yes

**□** No

**23.Is digital rectal examination a screening method for rectal cancer?**

**□** Yes

**□** No

**24. Is colonoscopy the gold standard for colorectal cancer screening?**

**□** Yes

**□** No

1. **Which of the following do you think can help prevent colorectal cancer? (Multiple choices allowed)**

**□** Colonoscopy

**□** Exercise

**□** Mood management

**□** Diet modification

**□** None of the above
