# Supplementary material for: Colorectal cancer screening awareness among rural populations in Northern China
Source: BMC Public Health. 2026 Jul 28;26:2234. doi: 10.1186/s12889-026-28732-z (PMC13418789; doi:10.1186/s12889-026-28732-z)
Supplement: Supplementary file 2 — Supplementary Material 2. [file 12889_2026_28732_MOESM2_ESM.docx]

**Table S1 Study participants'** **risk factors related colorectal cancer (N=2091)**

| **Variable** |  | **N (%)** |
| --- | --- | --- |
| **Family history of CRC in first-degree relatives (parents, siblings, children)** | **Yes** | 15 (0.7) |
|  | **No** | 2075 (99.2) |
|  | **Missing** | 1 (0.1) |
| **Personal history of colorectal polyps** | **Yes** | 9 (0.4) |
|  | **No** | 2075 (99.2) |
|  | **Missing** | 7 (0.3) |
| **Chronic diarrhea ^a^** | **Yes** | 40 (1.9) |
|  | **No** | 2050 (98.0) |
|  | **Missing** | 1 (0.1) |
| **Chronic constipation ^b^** | **Yes** | 83 (4.0) |
|  | **No** | 2007 (96.0) |
|  | **Missing** | 1 (0.1) |
| **History of mucus and/or blood in stool** | **Yes** | 32 (1.5) |
|  | **No** | 2057 (98.4) |
|  | **Missing** | 2 (0.1) |
| **History of chronic appendicitis or appendectomy** | **Yes** | 45 (2.2) |
|  | **No** | 2044 (97.8) |
|  | **Missing** | 2 (0.1) |
| **History of chronic cholecystitis or cholecystectomy** | **Yes** | 65 (3.1) |
|  | **No** | 2014 (96.3) |
|  | **Missing** | 12 (0.6) |
| **History of adverse life events in the past 20 years that caused emotional trauma or distress** | **Yes** | 68 (3.3) |
|  | **No** | 1991 (95.2) |
|  | **Missing** | 32 (1.5) |
| **Positive FOBT (Fecal Occult Blood Test)** | **Yes** | 2 (0.1) |
|  | **No** | 86 (4.2) |
|  | **Undetected** | 1992 (95.2) |
|  | **Missing** | 11 (0.5) |

a Defined as diarrhea lasting a cumulative total of more than 3 months over the past 2 years, with each episode lasting more than 1 week. Diarrhea is a common symptom characterized by a significant increase in the frequency of bowel movements compared to usual habits (>3 times per day), loose or watery stools with a water content >85%, and a total daily stool weight exceeding 200 grams. It is often accompanied by a sense of urgency, anal discomfort, or incontinence.

b Defined as constipation lasting more than 2 months per year over the past 2 years. Constipation refers to reduced bowel movement frequency—fewer than 2–3 times per week, or only once every 2–3 days—with small, dry, and hard stools.

**Table S2 Colorectal cancer screening-related awareness among study participants (N=2091)**

| Variable |  | N (%) |
| --- | --- | --- |
| 1. Is advanced age a risk factor for colorectal cancer? | Yes | 1745 (83.5) |
|  | No | 345 (16.5) |
|  | Missing | 1 (0.0) |
| 2. Is lack of physical activity a risk factor for colorectal cancer? | Yes | 1582 (75.7) |
|  | No | 505 (24.2) |
|  | Missing | 4 (0.1) |
| 3. Is high consumption of beef, lamb, and processed meats a risk factor for colorectal cancer? | Yes | 1690 (80.8) |
|  | No | 400 (19.1) |
|  | Missing | 1 (0.0) |
| 4. Is smoking a risk factor for colorectal cancer? | Yes | 1702 (81.4) |
|  | No | 387 (18.5) |
|  | Missing | 2 (0.1) |
| 5. Is excessive alcohol consumption a risk factor for colorectal cancer? | Yes | 1743 (83.4) |
|  | No | 347 (16.6) |
|  | Missing | 1 (0.0) |
| 6. Is overweight or obesity a risk factor for colorectal cancer? | Yes | 1647 (78.8) |
|  | No | 439 (21.0) |
|  | Missing | 5 (0.2) |
| 7. Are colon polyps a risk factor for colorectal cancer? | Yes | 1843 (88.1) |
|  | No | 246 (11.8) |
|  | Missing | 2(0.1) |
| 8. Is inflammatory bowel disease a risk factor for colorectal cancer? | Yes | 1724 (82.5) |
|  | No | 361 (17.3) |
|  | Missing | 6 (0.3) |
| 9. Is a family history of colorectal cancer a risk factor for colorectal cancer? | Yes | 1826 (87.3) |
|  | No | 260 (12.4) |
|  | Missing | 5 (0.2) |
| 10. Is hereditary cancer syndrome a risk factor for colorectal cancer? | Yes | 1843 (88.1) |
|  | No | 245 (11.7) |
|  | Missing | 3 (0.1) |
| 11. Is diabetes a risk factor for colorectal cancer? | Yes | 1600 (76.5) |
|  | No | 485 (23.2) |
|  | Missing | 6 (0.3) |
| 12. Is the intake of dietary fiber, whole grains, and fermented dairy products (such as yogurt) a protective factor against colorectal cancer? | Yes | 1787 (85.5) |
|  | No | 297 (14.2) |
|  | Missing | 7 (0.3) |
| 13. Is rectal bleeding with bright red blood an early symptom of colorectal cancer? | Yes | 1801（86.1) |
|  | No | 287 (13.7) |
|  | Missing | 3 (0.1) |
| 14. Is blood in the stool, which might make the stool look dark brown or black, an early symptom of colorectal cancer? | Yes | 1848 (88.4) |
|  | No | 237 (11.3) |
|  | Missing | 6 (0.3) |
| 15. Is chronic diarrhea an early symptom of colorectal cancer? | Yes | 1802 (86.2) |
|  | No | 274 (13.1) |
|  | Missing | 15 (0.7) |
| 16. Is fatigue or anemia an early symptom of colorectal cancer? | Yes | 1618 (77.4) |
|  | No | 457 (21.9) |
|  | Missing | 16 (0.8) |
| 17. Is abdominal pain or a feeling of incomplete bowel evacuation an early symptom of colorectal cancer? | Yes | 1997 (95.5) |
|  | No | 49 (2.3) |
|  | Missing | 15 (0.7) |
| 18. Can early-stage colorectal cancer be asymptomatic? | Yes | 1729 (82.7) |
|  | No | 278 (13.3) |
|  | Missing | 84 (4.0) |
| 19. Is colonoscopy a screening method for colorectal cancer? | Yes | 1812 (86.7) |
|  | No | 267 (12.8) |
|  | Missing | 12 (0.6) |
| 20. Is the fecal occult blood test a screening method for colorectal cancer? | Yes | 1715 (82.0) |
|  | No | 360 (17.2) |
|  | Missing | 16 (0.8) |
| 21.Is a high-risk questionnaire a screening method for colorectal cancer? | Yes | 1659 (79.3) |
|  | No | 416 (19.9) |
|  | Missing | 16 (0.8) |
| 22. Is fecal genetic testing a screening method for colorectal cancer? | Yes | 1780 (85.1) |
|  | No | 293 (14.0) |
|  | Missing | 18 (0.9) |
| 23.Is digital rectal examination a screening method for rectal cancer? | Yes | 1655 (79.2) |
|  | No | 423 (20.2) |
|  | Missing | 13 (0.6) |
| 24. Is colonoscopy the gold standard for colorectal cancer screening? | Yes | 1702 (81.4) |
|  | No | 369 (17.7) |
|  | Missing | 20 (1.0) |
| 25. Which of the following do you think can help prevent colorectal cancer? (Multiple choices allowed) | Full score | 477 (22.8) |
|  | Half score | 1606 (76.8) |
|  | Missing | 8 (0.4) |

**Table S3** Comparison of study participants’ awareness score groups regarding their sociodemographic characteristics, special habits, and CRC-related risk factors

|  |  | **Awareness** | | ***p*-value (chi-square**  **test)** |
| --- | --- | --- | --- | --- |
|  |  | **Poor**  **(*N*=838)** | **Good（*N*=1253）** |  |
| **Age (years)** | **less than 18** | 9 (1.0) | 27 (2.1) | **<0.0001**** |
|  | **18-33** | 63 (7.5) | 147 (11.7) |  |
|  | **34-49** | 162 (19.3) | 290 (23.1) |  |
|  | **50-65** | 358 (42.7) | 462 (36.9) |  |
|  | **66 and above** | 246 (29.4) | 318 (25.4) |  |
| **Gender** | **Female** | 415 (49.5) | 661 (52.8) |  |
|  | **Male** | 423 (50.5) | 592 (47.2) | 0.1603* |
| **Marital status** | **Single** | 42 (5.0) | 100 (8.0) |  |
|  | **Married** | 723 (86.3) | 1083 (86.4) |  |
|  | **Divorced** | 13 (1.6) | 6 (0.5) |  |
|  | **Widow/widower** | 57 (6.8) | 64 (5.1) |  |
|  | **Others** | 3 (0.4) | 0 (0.0) | **0.0004**** |
| **Education level** | **No formal education** | 185 (22.1) | 328 (26.2) |  |
|  | **Primary level** | 392 (46.8) | 560 (44.7) |  |
|  | **Secondary level** | 166 (19.8) | 247 (19.7) |  |
|  | **College level** | 88 (10.5) | 133 (10.6) |  |
|  | **University/Tertiary level** | 7 (0.8) | 5 (0.4) | 0.146** |
| **Smoking** | **Yes** | 211 (25.2) | 202 (16.1) |  |
|  | **No** | 627 (74.8) | 1051 (83.9) | <**0.0001*** |
| **Alcohol** | **None drinker** | 571 (68.1) | 1055 (84.2) |  |
|  | **Occasionally and lightly** | 265 (31.6) | 192 (15.3) |  |
|  | **Frequently and heavily** | 2 (0.2) | 6 (0.5) | <**0.0001**** |
| **Exercise** | **Often** | 691 (82.5) | 1012 (80.8) |  |
|  | **Lacks** | 147 (17.5) | 241 (19.2) | 0.3586* |
| **Diabetes** | **Yes** | 62 (7.4) | 89 (7.1) |  |
|  | **No** | 775 (92.5) | 1160 (92.6) |  |
|  | **Others** | 1 (0.1) | 4 (0.3) | 0.7531** |
| **Do any of your immediate family members (parents, siblings, children) have a history of colorectal cancer?** | **Yes** | 5 (0.6) | 10 (0.8) |  |
|  | **No** | 833 (99.4) | 1243 (99.2) | 0.7868* |
| **Do you have a history of colorectal polyps?** | **Yes** | 5 (0.6) | 4 (0.3) |  |
|  | **No** | 833 (99.4) | 1249 (99.7) | 0.4976** |
| **Do you have a history of chronic diarrhea?** | **Yes** | 25 (3.0) | 15 (1.2) |  |
|  | **No** | 813 (97.0) | 1238 (98.8) | **0.005*** |
| **Do you have a history of chronic constipation?** | **Yes** | 50 (6.0) | 33 (2.6) |  |
|  | **No** | 788 (94.0) | 1220 (97.4) | **0.0002*** |
| **Do you have a history of mucus and/or blood in your stool?** | **Yes** | 5 (0.6) | 27 (2.2) |  |
|  | **No** | 833 (99.4) | 1226 (97.8) | **0.008*** |
| **Do you have a history of chronic appendicitis or appendectomy?** | **Yes** | 11 (1.3) | 34 (2.7) |  |
|  | **No** | 827 (98.7) | 1219 (97.3) | **0.044*** |
| **Do you have a history of chronic cholecystitis or cholecystectomy?** | **Yes** | 12 (1.4) | 53 (4.2) |  |
|  | **No** | 826 (98.6) | 1200 (95.8) | **0.0005*** |
| **Have you experienced any adverse life events in the past 20 years that caused emotional trauma or distress?** | **Yes** | 31 (3.7) | 37 (3.0) |  |
|  | **No** | 807 (96.3) | 1216 (97.0) | 0.4139* |
| **What were the results of your fecal occult blood test (immunological method)?** | **Yes** | 0 (0.0) | 2 (0.2) |  |
|  | **No** | 20 (2.4) | 66 (5.3) |  |
|  | **Others** | 818 (97.6) | 1185 (94.6) | **0.0009**** |

*Chi-squared test

**Fisher Exact test, Bold p-values indicating significance

**Table S4** **Sensitivity Analysis of Factors associated with CRC-screening poor awareness among study participants after Excluding Participants <18 Years (N=2046)**

|  |  | **B** | **Exp(B)** | **95% C.I for EXP(B)** | | ***p*-value** |
| --- | --- | --- | --- | --- | --- | --- |
|  |  |  |  | **Lower** | **Upper** |  |
| **Age (years)** | **18-33** |  |  |  |  |  |
|  | **34-49** | -0.680 | 0.507 | 0.322 | 0.797 | **0.003** |
|  | **50-65** | -1.052 | 0.349 | 0.219 | 0.557 | **0.001** |
|  | **66 and above** | -1.357 | 0.257 | 0.155 | 0.426 | **0.001** |
| **Gender** | **Female** |  |  |  |  |  |
|  | **Male** | 0.374 | 1.454 | 1.133 | 1.867 | **0.003** |
| **Marital status** | **Single** |  |  |  |  |  |
|  | **Married** | 0.523 | 1.687 | 0.934 | 3.045 | 0.083 |
|  | **Divorced** | -0.579 | 0.560 | 0.165 | 1.898 | 0.352 |
|  | **Widow/widower** | -0.117 | 0.778 | 0.541 | 2.333 | 0.754 |
|  | **Others** | -20.166 | 0.000 | 0.000 | -- | 0.999 |
| **Education level** | **No formal education** |  |  |  |  |  |
|  | **Primary level** | -0.327 | 0.721 | 0.551 | 0.944 | **0.045** |
|  | **Secondary level** | -0.382 | 0.683 | 0.490 | 0.952 | **0.024** |
|  | **College level** | -0.743 | 0.476 | 0.302 | 0.749 | **0.001** |
|  | **University/Tertiary level** | -1.720 | 0.179 | 0.049 | 0.659 | **0.010** |
| **Smoking consumption** | **Yes** | -0.292 | 0.746 | 0.546 | 1.021 | 0.067 |
|  | **No** |  |  |  |  |  |
| **Alcohol consumption** | **None drinker** |  |  |  |  |  |
|  | **Occasionally and lightly** | -0.791 | 0.454 | 0.335 | 0.613 | **0.001** |
|  | **Frequently and heavily** | 0.520 | 1.68 | 0.317 | 8.924 | 0.541 |
| **Exercise** | **Often** | -0.546 | 0.579 | 0.445 | 0.753 | 0.172 |
|  | **Lacks** |  |  |  |  |  |
| **Diabetes** | **Yes** | 0.114 | 1.121 | 0.782 | 1.605 | 0.535 |
|  | **No** |  |  |  |  |  |

B the unstandardized regression coefficient

Exp(B) the exponentiated coefficient, indicating the adjusted odds ratio

95% C.I. the 95% confidence interval
